# Insect ectoparasites on wild birds in the Czech Republic during the pre-breeding period

**DOI:** 10.1051/parasite/2011181013

**Published:** 2011-02-15

**Authors:** O. Sychra, I. Literák, P. Podzemný, P. Harmat, R. Hrabák

**Affiliations:** 1 Department of Biology and Wildlife Diseases, Faculty of Veterinary Hygiene and Ecology, University of Veterinary and Pharmaceutical Sciences Palackého 1-3 612 42 Brno Czech Republic

**Keywords:** chewing lice, Phthiraptera, fleas, Siphonaptera, birds, Passeriformes, spring migration, Czech Republic, mallophages, Phthiraptera, puces, Siphonaptera, oiseaux, Passériformes, migration printanière, République Tchèque

## Abstract

Wild passerine birds (Passeriformes) from the northeastern part of the Czech Republic were examined for ectoparasites during the pre-breeding period in 2007. Two species of fleas of the genera *Ceratophyllus* and *Dasypsyllus* (Siphonaptera: Ceratophyllidae), and 23 species of chewing lice belonging to the genera *Ricinus*, *Myrsidea*, *Menacanthus* (Phthiraptera: Menoponidae), *Brueelia*, *Penenirmus*, and *Philopterus* (Phthiraptera: Philopteridae) were found on 108 birds of 16 species. Distribution of insect ectoparasites found on wild birds during pre-breeding was compared with previous data from the post-breeding period. There was no difference in total prevalence of chewing lice in prebreeding and post-breeding periods. Higher prevalence of fleas and slightly higher mean intensity of chewing lice were found on birds during the pre-breeding period. There was a significant difference in total prevalence but equal mean intensity of chewing lice on resident and migrating birds.

## Abstract (fr)

Des oiseaux sauvages (Passériformes) ont été examinés pour la présence des ectoparasites dans la partie nord-est de la République Tchèque pendant la période qui précède la reproduction. Deux espèces de puces des genres *Ceratophyllus* et *Dasypsyllus* (Siphonaptera : Ceratophyllidae) et 23 espèces de mallophages appartennant aux genres *Ricinus*, *Myrsidea*, *Menacanthus* (Phthiraptera: Menoponidae), *Brueelia*, *Penenirmus* et *Philopterus* (Phthiraptera : Philopteridae) ont été recoltées sur 108 oiseaux de 16 espèces. La distribution des insectes ectoparasites sur les oiseaux sauvages recoltés en période avant la reproduction a été comparée avec les données précédentes de la période après la reproduction. Il n’a été observé aucune différence dans la prévalence des mallophages entre les périodes avant et après la reproduction. Une prévalence plus élevée des puces et une intensité moyenne légèrement plus élevée des mallophages ont été trouvées sur les oiseaux en période avant la reproduction. La différence entre les prévalences totales des mallophages sur les oiseaux sédentaires et migrateurs a été significative, tandis que les intensités moyennes ont été égales pour les deux.

## Introduction

Ectoparasites, including chewing lice, can impact upon body condition, fitness, ability to fly or even long-term survival of their hosts. The impact of ectoparasites on energetics may also be responsible for significant drop in the rate of male courtship display, and thus in the ability of heavily infested males to attract mates (see Price *et al.*, 2003 for review). On the other hand, almost nothing is known about the role of ectoparasites in bird migration, and in particular regarding small transcontinental passerine birds (Passeriformes). In a previous paper (Sychra *et al.*, 2008), we presented new data on the species richness and distribution of insect ectoparasites found on some passerine birds in the Czech Republic during the post-breeding period. The aims of this paper are: (1) to present new data on the distribution of insect ectoparasites found on some wild birds in the Czech Republic during pre-breeding migration; (2) to include information on their prevalence, intensity and abundance; and (3) to compare distribution of insect ectoparasites found on wild birds during pre-breeding and post-breeding periods.

## Materials and Methods

### Study Areas

Fieldwork was carried out in a forest aisle adjacent to pasture located in the Sub-Beskidian Hills, near Čert’ák (49°34’N, 17°59’E) at an elevation of about 400 m above sea level at the same place where we had worked in 2005 (Sychra *et al.*, 2008).

### Methods

Birds were examined during the main season of prebreeding migrations from 31 March to 28 April 2007. Birds were examined and chewing lice collected as described elsewhere (Sychra *et al.*, 2008). Identification of the lice was based on papers by Price (1977), Rheinwald (1968) and Zlotorzycka (1976, 1977). The nomenclature of the lice follows Price *et al.* (2003). Identification of the fleas was based on a paper by Rosický (1957). The taxonomy of the birds follows Dickinson (2003).

The following parasitological parameters are evaluated in this paper: (1) Richness is the number of species of ectoparasites on a host taxon; (2) Host specificity is the range of host taxa infested by a given ectoparasite taxon; (3) Dominance is the number of individuals of a parasite species as a percentage of the total number of individuals collected from examined birds; (4) Prevalence is the proportion of the members of a taxon infested with ectoparasites; (5) Mean intensity is number of individuals of a particular ectoparasite species on infested hosts; and (6) Mean abundance is number of individuals of a particular ectoparasite species on examined birds (Marshall, 1981; Bush *et al.*, 1997).

For purposes of statistical analysis, we divided the birds into two groups: resident and migrating. Included into the analysis were only species of birds with at least ten examined individuals. Resident birds are birds with no or only short-distance post-breeding movement. These included *Turdus merula*, *Emberiza citrinella*, *Parus major*, *Cyanistes caeruleus*, *Periparus ater* and *Coccothraustes coccothraustes*. Most of these birds usually overwinter in the Czech Republic (Cepák *et al.*, 2008). Migrating birds are birds with long-distance migration. These were *Turdus philomelos*, *Fringilla coelebs*, *Erithacus rubecula*, *Sylvia atricapilla*, *Phylloscopus collybita*, *Prunella modularis* and *Ficedula hypoleuca*. The majority of them overwinter in the Mediterranean, but some of them are even transcontinental migrants (Cepák *et al.*, 2008). Birds with extremely high numbers of chewing lice (one *Turdus merula* was parasitized with 444 specimens of *Brueelia merulensis*) or which were examined in extreme high number (*Erithacus rubecula*, n = 281 and *Sylvia atricapilla*, n = 114) were not included into the analysis.

## Results

A total of 659 individuals of 34 bird species belonging to 11 passerine families – Motacillidae, Sylviidae, Prunellidae, Turdidae, Muscicapidae, Troglodytidae, Paridae, Aegithalidae, Certhiidae, Fringillidae and Emberizidae – and one family of woodpeckers (Piciformes, Picidae), were examined during the pre-breeding migration. One hundred and eight birds (16.4%, n = 659) of 16 species were parasitized with two species of fleas of the genera *Ceratophyllus* Curtis and *Dasypsyllus* Baker, and 23 species of chewing lice of the genera *Ricinus* De Geer, *Myrsidea* Waterston, *Menacanthus* Neumann, *Brueelia* Kéler, *Penenirmus* Clay and Meinertzhagen and *Philopterus* Nitzsch (see Appendix 1). No species of louse fly (Hippoboscidae) was found. No chewing lice were found on *Dendrocopos minor* (1 specimen examined, Piciformes, Picidae) or on the following passerines from the following families: Muscicapidae: *Ficedula hypoleuca* (13), *F. albicollis* (1), *Phoenicurus phoenicurus* (3), *Luscinia megarhynchos* (1); Sylviidae: *Phylloscopus trochilus* (7), *Sylvia communis* (3), *S. curruca* (3), *Regulus regulus* (1), *R. ignicapillus* (1); Motacillidae: *Anthus trivialis* (1); Fringillidae: *Coccothraustes coccothraustes* (11), *Carduelis chloris* (1), *C. cardueli*s (1); Paridae: *Poecile montanus* (4), *P. palustris* (5); Aegithalidae: *Aegithalos caudatus* (4), and Certhiidae: *Certhia familiaris* (4).

The average number of ectoparasite species was 1.2 on individual bird species. Most birds were parasitized with only one species of ectoparasite (84%, n = 89). Fewer birds were parasitized with two (11 cases) or three species of lice (three cases). The highest number of insect ectoparasite species was found on *Turdus merula* (see Appendix 1). The highest number of lice – 444 specimens of *Brueelia merulensis* – also was found on this host. Mean species richness, mean intensity and prevalence are given in Table I. The mean host specificity score for chewing lice was 1.4 (range 1-4). The following dominance was found for five genera of lice (n = 1587): *Brueelia* (46%), *Philopterus* (34%), *Menacanthus* (13%), *Penenirmus* (4%), *Ricinus* (2%) and *Myrsidea* (0.3%). The overall sex ratio of lice was female-biased (male: female = 1 : 1.9; n = 641; χ2 = 64, p < 0.001). The overall age ratio of lice was immature-biased (adults: immatures = 1 : 1.5; n = 1587; χ2 = 59, p < 0.001).

**Table I.** Parasitological parameters of insect ectoparasites collected from wild passerine birds in the Czech Republic during pre-breeding and post-breeding periods.

	Chewing lice				Fleas	
	Post-breeding 2005^1^ (15 spp.)			Pre-breeding 2007 (23 spp.)	Post-breeding 2005^1^ (2 spp.)	Pre-breeding 2007 (2 spp.)
	Total	Young	Adults	Adults^2^	Total	Total
Prevalence in %^3^	15.3	16.4	5.4	13.5	0.8	2.9
Mean intensity	7.0	5.3	9.4	13.0^4^	1	1
Range	1–38	1–22	1–38	1–93^4^	1	1
Mean abundance^3^	1.1	0.9	0.5	2.4	0.008	2.9

Richness (range)^5^		0.6 (0–5)		1.0 (0–5)	–6	0.1 (0–1)
Percentage males		33.3 (162)^7^		34.2 (641)^7^	–6	36.8 (19)^7^
Percentage adults		57.9 (280)^8^		40.4 (1,587)^8^	–	–

1 Sychra *et al.,* 2008;

2 only adult birds were examined;

3 number of birds examined: pre-breeding, n = 659, post-breeding: total, n = 262, young birds, n = 92, adult birds, n = 170;

4 one *Turdus merula* parasitized with 444 specimens of *Brueelia merulensis* is not included;

5 number of host species: pre-breeding, n = 34, post-breeding, n = 36;

6 only two fleas on two birds were collected during post-breeding period;

7 number of adults;

8 number of lice for which age was assessed.

There was no difference in total prevalence of chewing lice in pre-breeding and post-breeding periods (Fisher’s exact test, p = 0.528). Higher prevalence of fleas (Fisher’s exact test, p = 0.053) and slightly higher total mean intensity of chewing lice (Mann-Whitney U test, p = 0.059) were found on birds during the pre-breeding period. The overall age ratio of lice differs in both periods, with an adult-biased ratio in the post-breeding period and immature-biased ratio in the pre-breading period.

While only adult birds were examined in the prebreeding period, a higher prevalence of chewing lice had been found on young birds in the post-breeding period (16.4%, n = 92 against 5.4%, n = 170; Fisher’s exact test, p = 0.011).

Twenty-two birds of ten species were examined in both years when the sampling was carried out: *Aegithalos caudatus* (1 bird), *Certhia familiaris* (1), *Cyanistes caeruleus* (1), *Poecile montanus* (1), *Poecile palustris* (3), *Prunella modularis* (3), *Pyrrhula pyrrhula* (1), *Sylvia atricapilla* (5), *Turdus merula* (5), *Turdus philomelos* (1). Eight of them were parasitized with chewing lice (Table II).

**Table II.** Individual birds examined in both post-breeding (2005) as well as pre-breeding periods (2007) and their chewing lice.

Host	2005	Chewing louse	2007	Chewing louse
*Turdus merula*	2 September	–	1 April	*P. turdi (21 ex)*
*Turdus merula*	13 September	–	2 April	*M. eurysternus (6 ex)*
*Turdus merula*	26 August		3 April	*P. turdi (1S ex) B. amsel (13 ex) B. merulensis (60 ex)*
*Turdus merula*	2 September	B. merulensis (1 ex) M. eurysternus (1 ex)	4 April	–
*Sylvia atricapilla*	2 August	–	23 April	*My. sylviae (1 ex)*
*Prunella modularis*	30 July	–	6 April	*P. modularis (41 ex)*
*Prunella modularis*	18 August	–	7 April	*P. modularis (48 ex)*
*Cyanistes caeruleus*	21 September	–	31 March	*M. sinuatus (6 ex)*

B. *=* Brueelia; M. *=* Menacanthus; My. *=* Myrsidea; P. *=* Philopterus.

Five species of birds were parasitized with chewing lice in both years when the sampling was carried out: *Sylvia atricapilla*, *Turdus merula*, *Turdus philomelos*, *Troglodytes troglodytes* and *Pyrrhula pyrrhula*. Except for *Turdus merula* and *Troglodytes troglodytes*, different species of lice and total prevalence of chewing lice were found in these birds in the pre-breeding and post-breeding periods (Table III).

**Table III.** Comparison of chewing lice found on birds of individual species in pre-breeding and post-breeding periods.

	Post-breeding period in 2005			Pre-breeding period in 2007	
	Prevalence^1^	Mean intensity		Prevalence^1^	Mean intensity
*Erithacus rubecula*	(D = 6)			(D = 43)^2^	
*Brueelia tristis*	0/15	–		1/281	3
*Menacanthus eurysternus*	0/15	–		5/281	1.2
*Philopterus rubeculae*	0/15	–		3/281	2

*Sylvia atricapilla*	(D = 30)			(D = 17)	
*Brueelia neoatricapillae*	1/78	1		0/114	–
*Brueelia tovornikae*	1/78	1		0/114	–
*Menacanthus curuccae*	1/78	1		0/114	–
*Menacanthus eurysternus*	3/78	2		0/114	–
*Myrsidea sylviae*	1/78	1		1/114	1

*Phylloscopus collybita*	(D = 2)			(D = S)	
*Menacanthus agilis*	0/6	–		3/32	1.7
*Penenirmus rarus*	0/6	–		3/32	9.3

*Fringilla coelebs*	(D = 3)			(D = S)	
*Menacanthus eurysternus*	0/7	–		1/31	1
*Ricinus fringillae*	0/7	–		3/31	4.7

*Parus major*	(D = 3)			(D = 4)	
*Brueelia weberi*	0/7	–		1/28	1
*Menacanthus sinuatus*	0/7	–		11/28	5.4

*Prunella modularis*	(D = 4)			(D = 4)	
*Menacanthus eurysternus*	0/10	–		1/23	1
*Philopterus modularis*	0/10	–		8/23	37.5

*Turdus merula*	(D = S)			(D = 3)	
*Brueelia amsel*	0/12	–		2/21	17
*Brueelia merulensis*	7/12	4.7		10/21	15.3^3^
*Menacanthus eurysternus*	3/12	3.3		3/21	21.3
*Philopterus turdi*	1/12	4		7/21	13.6
*Ricinus elongatus*	0/12	–		3/21	3.3

*Turdus philomelos*	(D = 6)			(D = 2)	
*Brueelia merulensis*	5/15	1.8		1/15	58
*Philopterus turdi*	0/15	–		1/15	10

*Troglodytes troglodytes*	(D = 3)			(D = 1)	
*Penenirmus albiventris*	2/7	ll.5		2/7	7

*Pyrrhula pyrrhula*	(D = 1)			(D = 0.3)	
*Brueelia pyrrhularum*	1/3	9		0/2	–
*Philopterus citrinelae*	0/3	–		1/2	6

1 number of birds parasitized/number of birds examined;

2 dominance of the individual bird species in %;

3 one *Turdus merula* parasitized with 444 specimens of *Brueelia merulensis* is not included.

There was significant difference in total prevalence of chewing lice on resident and migrating birds (Fisher’s exact test, p < 0.001; Table IV). Prevalence on resident birds ranged from 39% to 100% (no lice were found on *Coccothraustes coccothraustes*, n = 11). Prevalence on migrating birds ranged from 0.8% to 39% (no lice were found on *Ficedula hypoleuca*, n = 13). On the other hand, mean intensity of chewing lice on resident and migrating birds was found to be equal (Mann-Whitney U test, p > 0.01). The overall sex ratio of lice was female-biased in both resident birds (male: female = 1 : 2.2; n = 298; χ2 = 5.9, p < 0.05) as well as migrating birds (male: female = 1 : 1.7; n = 98; χ2 = 42, p < 0.001). The overall age ratio of lice was equal for resident birds (adults: immatures = 1 : 0.9; n = 580; χ2 = 0.4, p > 0.01) but immature-biased for migrating birds (adults: immatures = 1 : 3.3; n = 417; χ2 = 117, p < 0.001).

**Table IV.** Parasitological parameters of chewing lice collected from resident and migrating birds during pre-breeding period in 2007. Birds with extreme high number of chewing lice (one *Turdus merula* parasitized with 444 specimens of *Brueelia merulensis*) or with extreme high number of examined birds *(Erithacus rubecula,* n = 281 and *Sylvia atricapilla,* n = 114) are not included.

	Chewing lice	
	Migrant (n = 114)^1^	Resident (n = 94)^2^
Prevalence (%)	18.4	50.0
Mean intensity (range)	19.9 (1–93)	12.3 (1–88)
Mean abundance	3.7	6.2
Richness (range)	1.6 (0–2)^1^	2.2 (0–5)^2^
Percentage males	37.8 (98)^3^	31.2 (298)^3^
Percentage adults	23.5 (417)^4^	51.4 (580)^4^

1 five species: *Turdus philomelos*, *Fringilla coelebs*, *Phylloscopus collybita*, *Prunella modularis*, *Ficedula hypoleuca*;

2 six species: *Turdus merula*, *Emberiza citrinella*, *Parus major*, *Cyanistes caeruleus*, *Periparus ater*, *Coccothraustes coccothraustes*;

3 number of adults;

4 number of lice for which age was assessed.

## Discussion

Louse flies had been commonly found on birds in central Europe during their post-breeding migration (Sychra *et al.*, 2008). Because of their life cycle, no louse flies were found on wild birds during their pre-breeding migration. This fact explains the marked difference in total insect parasite prevalence among birds examined in spring and in autumn (16.4% against 31.3%).

We found higher prevalence of fleas, *Ceratophyllus gallinae* and *Dasypsyllus gallinulae*, in birds during the pre-breeding period. Bird fleas usually overwinter in the larval or pupal stages away from the birds’ bodies, generally in bird nests but possibly also on the ground below the old nests. Adult fleas then remain in the cocoon until stimulated to hatch by, for example, warmth, exhaled carbon dioxide and vibration caused by birds when they start breed in the nest (Rosický, 1957) or when birds are moving near to cocoons on the ground. Birds searching for food on the ground can be infected by freshly hatched fleas. That is why the finding of fleas on the bodies of birds is more likely in spring than in autumn.

The prevalence and intensity of chewing lice recorded in the present study are basically the same as those recorded in the post-breeding period (Sychra *et al.*, 2008). However, there is a slight tendency toward higher mean intensity of infestation on birds during the pre-breeding period. Prevalence of chewing lice as permanent ectoparasites is significantly influenced by the possibilities they have to spread to new hosts. In the breeding period, the most frequent is the horizontal route, *i.e.* transfer between two adult birds during, *e.g.*, mating (Price *et al.*, 2003). Chewing lice populations are known to grow in size in spring as a result of the onset of their hosts’ breeding period. This may be the explanation for slightly higher bird infestation with chewing lice in spring. The vertical chewing lice transfer route, *i.e.* from adults to young, is activated during the nesting period. Thanks to that, the chewing lice prevalence usually decreases on adults and conversely increases on young birds during this period (Price *et al.*, 2003). The differences ascertained in chewing lice prevalence on young and adult birds seem to corroborate these assumptions.

Prior to migration, birds’ condition improves and birds in good condition may control larger amounts of parasites more easily (Marshall, 1981; Price *et al.*, 2003).In accordance with these assumptions, chewing lice abundance before migration is low. The energy cost of migration is very high for birds, and, when they reach their wintering sites, the birds must therefore spend more time on feeding at the cost of other activities, including preening, which may result in an increase in chewing lice abundance (Rózsa, 1997; Price *et al.*, 2003). External conditions of wintering sites may be considerably different from those at the birds’ nest sites. These different conditions, and higher relative air humidity at wintering sites in particular, may have a positive effect on chewing lice abundance. Species occurring over a large territory are known to harbor larger chewing lice populations in areas with higher ambient humidity than in arid areas, which is also true for individual birds of the same species (Fabiyi, 1996; Moyer *et al.*, 2002). In the post-nesting period, a number of avian species also change their social behavior. During this period, the strictly territorial species tend to gather in flocks where more frequent contacts between individual birds are likely (Hudec, 1983). Such a situation, too, acts to favor higher chewing lice abundance (Rózsa, 1997). It may therefore be assumed that chewing lice abundance increases among birds at wintering sites. However, there are almost no studies to date into parasite incidence among migratory avian species. The findings of Brooke & Nakamura (1999) seem to indicate that that assumption might be correct.

The major factors that might explain marked differences in chewing lice incidence figures in resident and migratory bird species are: (1) the dynamics of chewing lice populations, and (2) the fact of bird migration. With regard to the first of these factors, at the time of year when the sampling was carried out (April) most of the resident species are already nesting (Hudec, 1983). It is therefore possible that the increase in parasite abundance comes earlier in resident birds than in migratory species, because the latter are only on their way to their nesting sites at that time. Foster (1969) had mentioned that higher occurrence of chewing lice on adult birds during the breeding period may be influenced by the production of reproductive hormones. Photoperiod is another factor that plays a crucial role in the timing of birds’ breeding seasons, and especially for resident birds (Perrins, 1970). A question is whether photoperiod also can play a role in the timing of chewing lice breeding.

Some chewing lice species are known to survive their hosts’ migration period as eggs (Price *et al.*, 2003). The majority of chewing lice species prefer to lay eggs in those areas on the hosts’ bodies where they will be best protected against the hosts’ preening, *i.e.* on the neck or between feather shafts (Price *et al.*, 2003). If that is the case, then chewing lice nymphs should outnumber adult chewing lice at the time when birds arrive to their nesting sites. The differences found in nymphs-toadults ratios in populations of migratory and resident birds seem to corroborate that assumption. With regard to the second factor mentioned above, migration affects the overall condition of migrating birds, and the higher numbers of ectoparasites, including chewing lice, may negatively affect the hosts’ chances for safe return to the nesting site. Birds in poor condition or those with higher parasite loads may have already perished in the early stages of the migration (Cork *et al.*, 2001). In their multi-year study of cliff swallows (*Petrochelidon pyrrhonota*), Brown *et al.* (1995) found that chewing lice together with other ectoparasites may significantly influence the return rate of parasite-infested birds from wintering sites to their nesting sites.

The very low prevalence of chewing lice on *Erithacus rubecula* (0.4–1.8%) and *Sylvia atricapilla* (0.8%), the two hosts with the highest number of individuals examined, seem to corroborate that assumption. The overall prevalence of chewing lice on *E. rubecula* was 3.6% (n = 281), however, which prevalence was quite similar to the 7% (n = 14) for birds examined in England and 0% (n = 49) for birds examined in Sweden as recorded by Ash (1960). That author had mentioned that chewing lice usually occur throughout the entire range of their hosts, but that should they for some reason not occur in a certain locality their establishment there is likely to be extremely slow. Aside from local distribution of chewing lice, there can be different rates of infestation also between years. During the course of the present work, some birds that had been trapped uninfested had later been retrapped and found to be infested (Table II). They could either have acquired chewing lice from some other individual of the same species or the chewing lice may have occurred in very low intensity on these birds and thus been overlooked during earlier examinations (see also Ash, 1960).

There could be many factors influencing the occurrence, prevalence and mean intensity of infestation by ectoparasites. Our results indicate that ectoparasites, including chewing lice, may play a role in bird migration, and especially in small transcontinental passerine birds. More data and experimental surveys are needed to resolve how important is this phenomenon for wild bird populations.

## Appendix 1.

**Appendix 1.** List of bird hosts and their insect ectoparasites in the Czech Republic during the pre-breeding migration.

Bird species	Prevalence	Ectoparasite family/species	♂^1^	♀^2^	Imm^3^
Family Picidae					
*Dendrocopos major* (Linnaeus, 1758)	3/4	P/*Brueelia straminea (Denny, 1842)**	14	22	12
	4/4	P/*Penenirmus auritus (Scopoli, 1763)*	6	8	6

Family Sylviidae					
*Sylvia atricapilla* (Linnaeus, 1758)	1/114	M/*Myrsidea sylviae* Sychra & Literák 2008	1		
	1/114	C/*Ceratophyllus gallinae* (Schrank, 1803)*		1	
*Phylloscopus collybita* (Vieillot, 1817)	3/32	M/*Menacanthus agilis* (Nitzsch, 1866)		4	1
	3/32	P/*Penenirmus rarus* (Zlotorzycka, 1976)	3	4	21
	1/32	C/*Dasypsyllus gallinulae (Dale*, 1878)		1	

Family Prunellidae					
*Prunella modularis* (Linnaeus, 1758)	1/23	M/*Menacanthus eurysternus (Burmeister, 1838)**			1
	8/23	P/*Philopterus modularis (Denny, 1842)*	23	35	242

Family Muscicapidae					
*Erithacus rubecula (Linnaeus, 1758)*	5/281	M/*Menacanthus eurysternus (Burmeister, 1838)**		6	
	1/281	P/*Brueelia tristis (Giebel, 1874)*		2	1
	3/281	P/*Philopterus rubeculae (Denny, 1842)**	2	3	1
	8/281	C/*Dasypsyllus gallinulae (Dale, 1878)*	3	5	
	5/281	C/*Ceratophyllus gallinae (Schrank, 1803)*	3	2	

Family Turdidae					
*Turdus merula Linnaeus, 1758*	3/21	R/*Ricinus elongatus (Olfers, 1816)*		7	3
	3/21	M/*Menacanthus eurysternus (Burmeister, 1838)*	3	13	48
	2/21	P/*Brueelia amsel (Eichler, 1951)**	7	16	11
	10/21	P/*Brueelia merulensis (Denny, 1842)*	74	150	358
	7/21	P/*Philopterus turdi (Denny, 1842)*	17	39	39
	1/21	C/*Dasypsyllus gallinulae (Dale, 1878)**		1	
*Turdus philomelos Brehm, C. L., 1831*	1/15	P/*Brueelia merulensis (Denny, 1842)*	8	7	43
	1/15	P/*Philopterus turdi (Denny, 1842)*	1	7	2
*Turdus viscivorus Linnaeus, 1758*	1/1	P/*Philopterus vernus (Zlotorzycka, 1964)*	1	1	6

Family Troglodytidae					
*Troglodytes troglodytes (Linnaeus, 1758)*	2/7	P/*Penenirmus albiventris (Scopoli, 1763)*		9	5

Family Paridae					
*Periparus ater (Linnaeus, 1758)*	5/11	M/*Menacanthus sinuatus (Burmeister, 1838)*	4	4	13
*Cyanistes caeruleus (Linnaeus, 1758)*	5/13	M/*Menacanthus sinuatus (Burmeister, 1838)*	10	19	25
	2/13	C/*Ceratophyllus gallinae (Schrank, 1803)*	1	1	
*Parus major Linnaeus, 1758*	11/28	M/*Menacanthus sinuatus (Burmeister, 1838)*	9	15	35
	1/28	P/*Brueelia weberi Balat, 1982*		1	
		C/*Ceratophyllus gallinae (Schrank, 1803)*		1	

Family Fringilidae					
*Fringilla coelebs Linnaeus, 1758*	1/28	R/*Ricinus fringillae De Geer, 1778*	2	3	9
	1/31	M/*Menacanthus eurysternus (Burmeister, 1838)*		1	
*Loxia curvirostra Linnaeus, 1758*	1/1	M/*Myrsidea quadrimaculata Carriker, 1902**		1	2
	1/1	P/*Philopterus curvirostrae (Schrank, 1776)*	11	7	9
*Pyrrhula pyrrhula (Linnaeus, 1758)*	1/2	P/*Philopterus citrinellae (Schrank, 1776)*	1	1	4

Family Emberizidae					
*Emberiza citrinella Linnaeus, 1758*	2/11	R/*Ricinus fringillae De Geer, 1778*	1	3	
	1/11	M/*Menacanthus alaudae (Schrank, 1776)**	2	1	
	10/11	P/*Philopterus citrinellae (Schrank, 1776)*	17	32	48

Prevalence = number of birds parasitized/number of birds examined; C = Ceratophyllidae; R = Ricinidae; M = Menoponidae; P = Philopte- ridae;

* first record of the host-parasite association in the Czech Republic;

^1^, ^2^, ^3^ numbers of males, females and immatures, respectively.
